# Metal Homeostasis Regulators Suppress FRDA Phenotypes in a *Drosophila* Model of the Disease

**DOI:** 10.1371/journal.pone.0159209

**Published:** 2016-07-19

**Authors:** Sirena Soriano, Pablo Calap-Quintana, José Vicente Llorens, Ismael Al-Ramahi, Lucía Gutiérrez, María José Martínez-Sebastián, Juan Botas, María Dolores Moltó

**Affiliations:** 1 Department of Genetics, University of Valencia, Burjassot, Valencia, Spain; 2 Department of Molecular and Human Genetics, Baylor College of Medicine, Houston, Texas, United States of America; 3 Department of Biomaterials and Bioinspired Materials, Instituto de Ciencia de Materiales de Madrid/CSIC, Madrid, Spain; 4 CIBERSAM, INCLIVA, Valencia, Spain; Sant Joan de Déu Children's Hospital, SPAIN

## Abstract

Friedreich’s ataxia (FRDA), the most commonly inherited ataxia in populations of European origin, is a neurodegenerative disorder caused by a decrease in frataxin levels. One of the hallmarks of the disease is the accumulation of iron in several tissues including the brain, and frataxin has been proposed to play a key role in iron homeostasis. We found that the levels of zinc, copper, manganese and aluminum were also increased in a *Drosophila* model of FRDA, and that copper and zinc chelation improve their impaired motor performance. By means of a candidate genetic screen, we identified that genes implicated in iron, zinc and copper transport and metal detoxification can restore frataxin deficiency-induced phenotypes. Taken together, these results demonstrate that the metal dysregulation in FRDA includes other metals besides iron, therefore providing a new set of potential therapeutic targets.

## Introduction

Friedreich’s ataxia (FRDA) is a neurodegenerative disorder caused by an intronic GAA expansion within *FXN*, the gene encoding frataxin [1]. Unstable GAA expansions in both alleles of this gene [2] inhibit transcription, therefore causing a reduction in frataxin protein levels [3,4]. FRDA is the most commonly inherited ataxia among populations of European origin [5] and is characterized by progressive gait and limb ataxia, tendon areflexia, muscle weakness and peripheral sensory neuropathy occurring at early adulthood. Non-neurological manifestations include hypertrophic cardiomyopathy leading to morbidity, diabetes mellitus or carbohydrate intolerance [6]. Affected individuals are wheelchair-bound during later stages of disease, and early death may occur at the fourth to fifth decade of life.

Frataxin deficiency results in several biochemical disturbances including impaired iron-sulphur cluster biogenesis, dysfunction of respiratory chain complexes and aconitase, accumulation of mitochondrial iron coupled to cytosolic iron depletion and increased oxidative stress sensitivity (reviewed in [7]). Although frataxin function is not fully elucidated, it is accepted that frataxin is critical for iron homeostasis [8] and marked accumulation of iron has been reported in several tissues of FRDA patients. Iron appears to accumulate in myocardium [9–11], and in liver and spleen as well as in the dentate nucleus of the cerebellum [9,12]. Iron chelation was therefore promptly proposed as therapeutic approach for the disease and data from pre-clinical studies [13,14] were promising. However, early-phase clinical trials with the iron chelator deferiprone have not lead to conclusive results and indicate a possible improvement only in some aspects of FRDA pathogenesis [15,16].

In addition to iron, redistribution of copper and zinc was also described in the dentate nucleus of FRDA patients [17], as well as changes in the cellular localization of the zinc transporter Zip14 in the dorsal root ganglia [18]. These findings presented a novel perspective of FRDA pathophysiology by suggesting that metal dysregulation extends beyond iron accumulation. Interestingly, there is extensive evidence of metal content imbalance in other neurodegenerative disorders such as Alzheimer’s (AD), Parkinson’s and Huntington’s diseases as well as amyotrophic lateral sclerosis. Copper, zinc, aluminum and manganese appear to play an important role in these pathologies, and may induce oxidative stress, protein misfolding and aggregation or neuroinflamation, leading eventually to neurodegeneration (reviewed in [19]).

Based on the potential role of metal imbalance in FRDA pathogenesis, we set out to test whether genetic modification of key pathways regulating metal content and distribution would improve FRDA phenotypes by restoring metal homeostasis. To test a broad range of components within these pathways in a high-throughput manner, we utilized *Drosophila* as a model of FRDA [20–22] in a genetic screen of potential modifiers. By focusing on a candidate pathway screening approach, we report several novel genetic modifiers of eye morphology and motor dysfunction in the FRDA fly model. These data provide further support for the notion that disruptions in metal homeostasis may be a primary contributor to FRDA disease pathogenesis.

## Materials and Methods

### *Drosophila melanogaster* strains

Two lines that knockdown *fh*, the homolog of *FXN* in *Drosophila*, were used in this study: (i) UDIR2 [21], from Dr. Phillips from University of Guelph, Canada, and (ii) UAS-*fh*IR [22] previously generated in our laboratory. Both lines have been renamed in the text and figures as *fh*RNAi-1 and *fh*RNAi-2 respectively, as in [23]. When expressed ubiquitously, *fh*RNAi-1 induced a strong interference with a 90% reduction of frataxin expression that resulted in adult lethality, whereas *fh*RNAi-2 produced a 70% reduction compatible with a normal development. *w*^*1118*^ strain was used as control in all the experiments. The driver lines *GMR*-GAL4 and *actin*-GAL4, which promote expression in the eye and in a ubiquitous pattern respectively were obtained from the Bloomington Stock Center (BSC, Indiana University, http://flystocks.bio.indiana.edu). For the genetic screen, we used shRNA lines from the Vienna Drosophila Resource Center (VDRC, http://stocks.vdrc.at) and loss-of-function and overexpression lines from BSC, except for tub-MTF-1 [24] that was kindly provided by Dr. Burke from University of Monash, Australia. A total of 130 lines from the VDRC and BSC selected from genes in metal homeostasis pathways were used to identify genetic modifiers of the phenotypes caused by frataxin knockdown in *Drosophila*.

### Culture conditions, metal chelation and climbing assay

*Drosophila* stocks were maintained at 25°C on standard cornmeal agar medium. The standard medium was supplemented with the copper chelators Tetrathiomolybdate (TTM) at 10 μM dissolved in 0.1% dimethylsulfoxide (DMSO) and Bathocuproine disulphonate (BCS) at 300 μM in H_2_O, and with the zinc chelator N,N,N',N'-tetrakis (2-pyridinylmethyl)- 1,2- ethanediamine (TPEN) at 100 μM in EtOH/PBS. All chemicals were purchased from Sigma-Aldrich. Crosses were conducted at 25°C in the supplemented medium or the vehicle medium containing the dissolving agent but not the chelator. The F_1_ adults of appropriate genotype were transferred to fresh vials with the supplemented or vehicle medium every two days. We evaluated the effect of the metal chelators on the climbing ability as described in [25].

### Genetic screen

A total of 130 candidate lines from the VDRC and BSC selected for genes in metal homeostasis pathways were used to identify genetic modifiers of the external eye structure and the motor performance phenotypes induced by frataxin knockdown in *Drosophila*. For the eye screen, UAS-*fh*RNAi-1; *GMR*-GAL4 flies were crossed at 27°C with the candidate lines, and the external eye structure of the F_1_ flies with appropriate genotype was observed with a Nikon light microscope. For the motor performance assays, we used the UAS-*fh*RNAi-2; *actin*-GAL4 line and both the crosses and the experimental individuals were maintained at 28°C. These assays were performed as previously described in [26]. We recorded the number of flies that climbed to a height of 11.5 cm after 16 s and the mean percentage of 10 observations was plotted for each day. Two replicates of 15 individuals were tested in parallel for each genotype.

### Measurement of metals and MDA + HAE levels

Inductively Coupled Plasma—Atomic Emission Spectroscopy (ICP-AES) was used to measure the levels of iron, zinc, copper, manganese and aluminum calibrated against standard solutions in an OPTIME 2100DV from Perkin Elmer. Previously, whole flies were lyophilized, weighed and digested with nitric acid (1 h at 90°C) and hydrogen peroxide (1h at 90°C). These assays were conducted in triplicate with 600 adult males per replicate. The concentration of malondialdehyde (MDA) + 4-hydroxyalkenals (HAE) was determined as described in [25]. MDA+HAE levels were normalized to the protein amount determined by the Bradford assay.

### Superoxide dismutase 1 (SOD1) activity assay

Cu,Zn-SOD1 activity was measured following the procedures described by the SOD Assay Kit (Sigma-Aldrich). Three replicates of 15 males for each condition (control or *fhRNAi-2* flies in vehicle or chelator) were homogenized in PBS 50 mM pH 7.4, EDTA 1 mM buffer and the supernatant was collected after a 1500 g, 5 min centrifugation at 4°C. A second centrifugation at 10000 g for 10 min was performed and the supernatant collected to obtain the cytosolic fraction, which was used to determine the SOD activity.

### Quantitative real-time PCR (RT-qPCR)

Total RNA was isolated from 50 males using RNeasy Minikit (Qiagen). RNA (2.5 μg), primed by oligo(dT)_20_-primers, was reverse transcribed with Expand Reverse Transcriptase (Roche Diagnostics). Quantitative PCR was carried out using Step One Plus Real-Time PCR System (Applied Biosystems) and Power SYBR Green (Applied Biosystems). Primer performance was assayed with a dilution series of cDNA. The primers used for transcript amplification of the *frataxin homolog* gene (*fh*) were: forward 5′- ACA CCC TGG ACG CAC TGT-3′, reverse 5′- CCA GGT TCA CGG TTA GCA C-3′. The house-keeping *Ribosomal protein 49* gene (*Rp49*) was used to normalize data (forward-primer 5′-CCG CTT CAA GGG ACA GTA TCT G-3′, reverse-primer 5′-CAC GTT GTG CAC CAG GAA CTT-3′). The relative quantification of each cDNA was calculated in quadruplicate experiments using the comparative Ct method.

### Statistical analysis

All statistical analyses were carried out with the GraphPad Prism 6 software. For comparison of means, we performed unpaired nonparametric Student's t test or one-way ANOVA followed by Dunnett or Sidak tests for multiple comparisons. In all cases, values of P<0.05 were considered statistically significant and errors bars represent the standard error of the mean (SEM) between independent experiments.

## Results and Discussion

### Frataxin deficiency in *Drosophila* leads to metal accumulation

**Fig 1:**
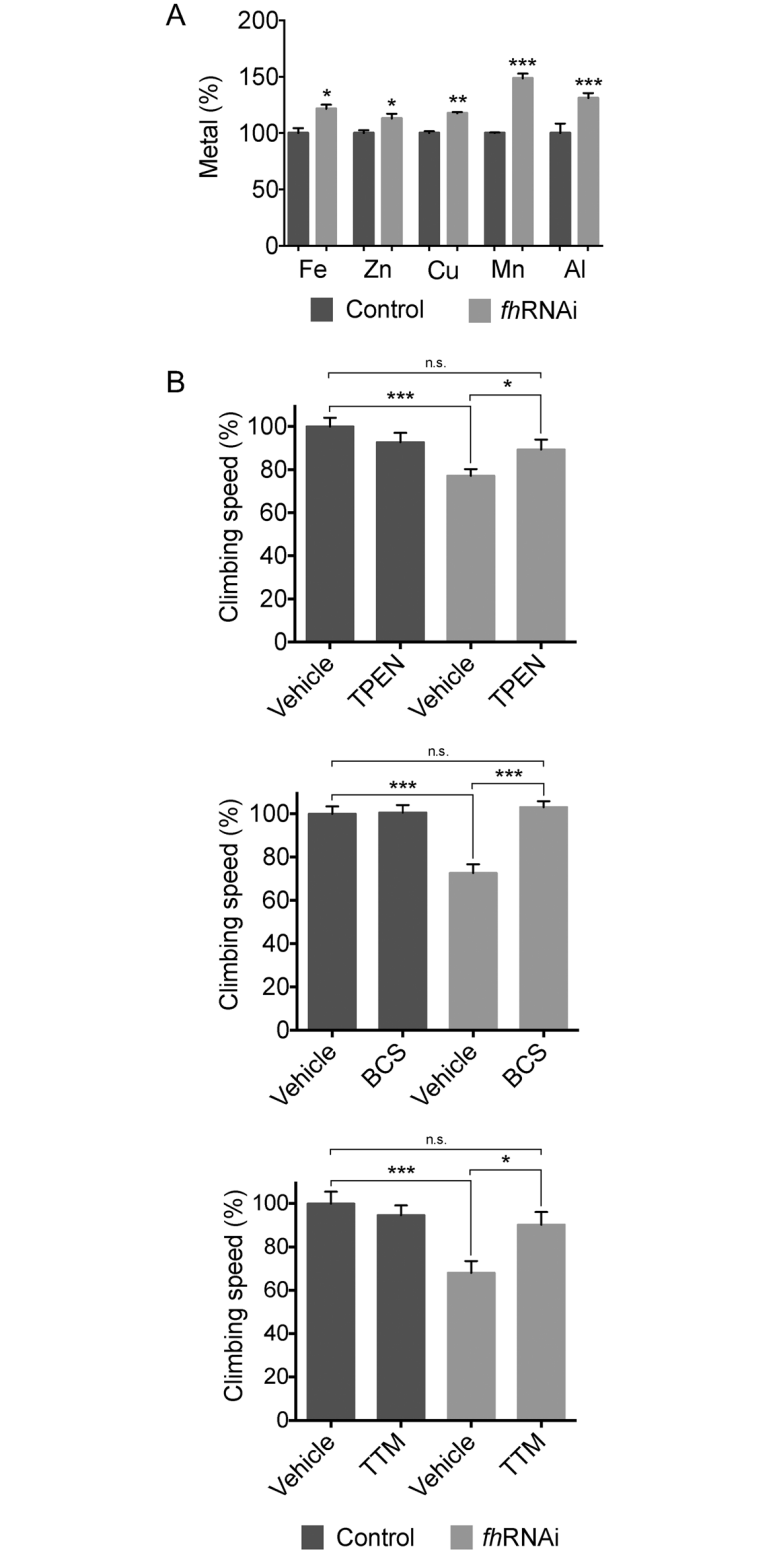
Metal accumulation in FRDA flies. A. Total iron, zinc, copper, manganese and aluminum levels measured by atomic emission spectroscopy and expressed in percentage relative to the controls are increased in *fh*RNAi flies (*actin*-GAL4>UAS-*fh*RNAi-2) *vs* controls (w^1118^; *actin*-GAL4). Statistical significance was evaluated by unpaired nonparametric Student's t test. B. Zinc chelator TPEN and copper chelators BCS and TTM improve the climbing ability of *fh*RNAi flies (*actin*-GAL4>UAS-*fh*RNAi-2) compared to vehicle medium (EtOH/PBS, H_2_O and DMSO 0.1%, respectively). The results are expressed in percentage, taking as 100% the mean climbing speed of control flies in the vehicle medium. Statistical significance was evaluated by ANOVA followed by Sidak test for multiple comparisons. n.s.: non-significant, *P<0.05, **P<0.01, ***P<0.001. Error bars represent SEM.

Iron accumulation occurs in several tissues of FRDA patients [9–12] and in models of disease [14,27–29]. Recently, copper and zinc dysregulation has also been suggested in FRDA [17,18]. Therefore, we hypothesized that ubiquitous reduction of frataxin driven by the *fh*RNAi-2 allele might cause a global alteration in metal content. Interestingly, atomic emission spectroscopic analysis revealed not only an increase in iron levels but that zinc, copper, manganese and aluminum were also increased in the FRDA flies relative to control animals (Fig 1A). Among these elements, Al is the only one that does not appear to have a known role in animal biology. This element is abundant in the environment and its uptake takes place via air, food and water. However, it has been suggested that Al can generate reactive oxygen species (ROS) [30] and its presence has been implicated in neurological disorders such as Alzheimer’s disease [19]. In addition, Wu et al. [31] found that our model of FRDA in *Drosophila* displays increased sensitivity to Al toxicity mediated by ROS production and Fe accumulation.

To investigate whether the increase in metal levels is implicated in the pathophysiology of FRDA, we asked whether metal chelation improves the climbing phenotype of the frataxin deficient flies reported by our laboratory [22]. Previously, we showed that the iron chelator deferiprone rescued several phenotypes of the FRDA fly model, including the motor impairment [14]. In this case, the zinc chelator TPEN was effective in inducing a significant recovery of the climbing ability of the FRDA animals (Fig 1B). The copper chelators TTM and BCS also ameliorated the climbing ability up to control levels (Fig 1B). Next, we measured the Fe content in the model flies when the availability of Zn or Cu was reduced by chelation. We found that the Fe levels were not affected by TPEN, TTM or BCS in our experimental conditions (S1 Fig). These results show that the beneficial effect of the chelators on the motor performance of the FRDA flies is not dependent on Fe levels and support that Zn and Cu also contributing to the frataxin deficient phenotype.

Because of the importance of metal cofactors for the key enzyme in antioxidant defence Cu,Zn-SOD1, we tested its activity after the treatment with the chelators. No significant changes in the enzyme activity were detected for the Zn chelator TPEN (S2 Fig) and the Cu chelator TTM (S2 Fig) at the doses tested. A significant reduction of the Cu,Zn-SOD1 activity was found with the Cu chelator BCS of in both the control flies and the FRDA flies (S2 Fig). Despite this reduction in the Cu,Zn-SOD1 activity, model flies showed a great improvement of their motor skills when they were treated with BCS (Fig 1B), suggesting that the beneficial effect of the Cu chelation is greater that the possible detrimental effect of the reduction in Cu,Zn-SOD1.

Taken together, these data indicate that the FRDA fly model recapitulates an imbalance in metal content, reminiscent of the human condition, and suggest that zinc and specially copper chelators might be of potential therapeutic interest for the treatment of the disease.

### Modifying key regulators of iron homeostasis suppresses FRDA fly phenotypes

To determine whether targeted alterations of pathways involved in metal homeostasis are sufficient to improve FRDA fly phenotypes, we systematically evaluated the effect of decreasing or increasing the expression of metal-associated genes using two different and independent assays in the FRDA *Drosophila* model. We reasoned that utilizing a tiered strategy of first evaluating modifiers of eye morphology followed by evaluating modifiers of motor performance would provide high confidence genetic modifiers of these disease phenotypes. For our primary screen of external eye morphology, we evaluated modifiers of a mild rough eye phenotype induced by specific expression of the *fh*RNAi-1 allele in the developing eye. We then used a milder frataxin knockdown system (*actin*-GAL4>UAS-*fh*RNAi-2, enables systemic frataxin reduction compatible with adult survival) to determine whether these modifiers were also able to improve the impairment in motor performance reported in FRDA flies [22].

**Fig 2:**
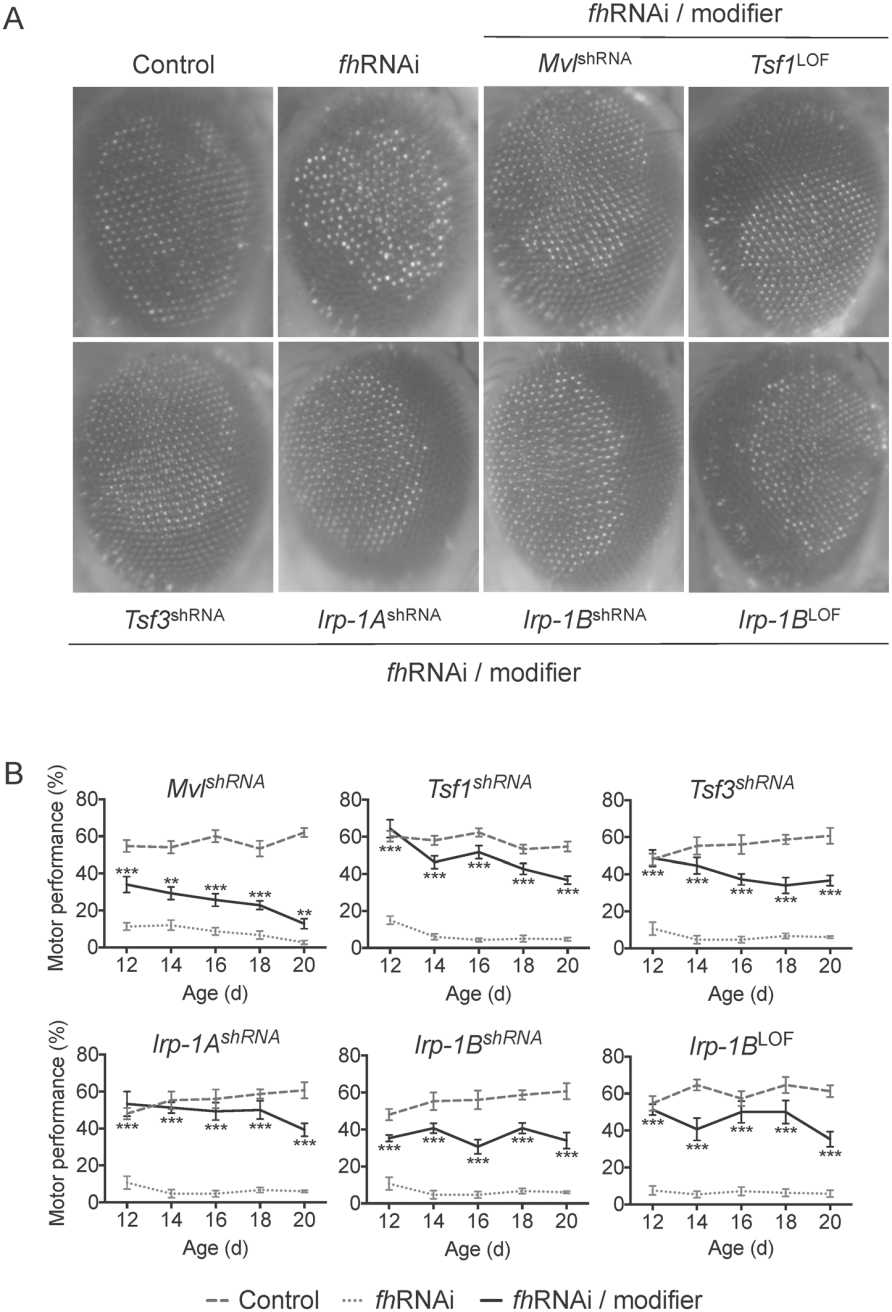
Improvement of the eye morphology and the motor performance phenotypes of FRDA flies by decreased expression of genes implicated in iron homeostasis. A. Light microscope images of the external eye of control (w^1118^; *GMR*-GAL4), *fh*RNAi (*GMR*-GAL4>UAS-*fh*RNAi-1) and *fh*RNAi/modifier flies (*GMR*-GAL4>UAS-*fh*RNAi-1 additionally carrying the corresponding allele of the modifier) B. Motor performance expressed as the percentage of flies that climbed passed a height of 11.5 cm. after 16 s. was evaluated at different adult ages, from 12 to 20 days old. Control: w^1118^; *actin*-GAL4. *fh*RNAi: *actin*-GAL4>UAS-*fh*RNAi-2. *fh*RNAi/modifier: *actin*-GAL4>UAS-*fh*RNAi-2; modifier allele. The statistical significance between *fh*RNAi and *fh*RNAi/modifier for each day was evaluated by ANOVA followed by Dunnet test for multiple comparisons. *P<0.05, **P<0.01, ***P<0.001. Error bars represent SEM. shRNA: knockdown allele. LOF: loss of function allele. d: day.

Given the central role of iron in FRDA pathogenesis, we first investigated whether genes implicated in iron homeostasis modify FRDA fly model phenotypes. Among the tested genes implicated in iron homeostasis, we found a total of 5 suppressors of both the eye and the motor performance phenotypes (Fig 2A and 2B). Genotypes of the *Drosophila* strains corresponding to these genetic interactors are shown in S1 Table.

Of the five suppressor genes, three genes function as iron carriers. Knockdown of Malvolio (*Mvl*), the *Drosophila* homolog of the mammalian *Divalent metal transporter-1* (*DMT1*) [32], improved both the eye and motor performance phenotypes caused by frataxin deficiency (Fig 2A and 2B). Similarly, genetic reduction of *Tsf1* and *Tsf3*, the *Drosophila* homologs of the iron transport carrier Transferrin [33], improved the phenotypes of the FRDA *Drosophila* model (Fig 2A and 2B). *Tsf1* is abundant in the fly hemolymph and there is evidence that it plays a role in immune response [34,35]. However whether it serves as an iron carrier between cells similarly to mammalian transferrin still remains unclear (reviewed in [36]). Even less is known about *Tsf3*, also homolog of transferrin, which has not been characterized yet. Interestingly, the identification of both *Tsf1* and *Tsf3* as suppressors for FRDA provides indirect evidence supporting their role in iron metabolism in *Drosophila*.

In addition to altering genes encoding iron carriers, we found that altering key regulators of iron homeostasis can also improve the eye morphology and motor performance phenotypes of FRDA model flies. Iron absorption and metabolism are regulated by the IRP/IRE system. When the cells are iron depleted, Iron Regulatory Proteins (IRPs) bind to Iron Responsive Elements (IREs) on the 5’UTR and 3’UTR mRNAs of their target proteins. Specifically, binding of IRPs inhibit the translation of the L and H chains of ferritin, the iron exporter FPN1 and the mitochondrial aconitase, among others, whereas it increases the transferrin receptor TfR1 and DMT1 (reviewed in [37]). Knocking down the homologs of the IRPs in *Drosophila*, *Irp-1A* and *Irp-1B* [38], in the FRDA model fly rescued the eye structure and the motor performance impairment (Fig 2A and 2B). An increase in IRP binding activity has been described in FRDA patient lymphoblasts and a cardiac mouse model [39,40], which is an indicator of a cytosolic iron depletion that contrasts with the mitochondrial iron overload observed in FRDA. *fh*RNAi-2 animals show a reduced expression of *Irp-1A* in iron overload conditions that has been suggested to represent a cellular response against the prolonged IRP binding [41]. Similarly, the reduction in gene expression of the *Drosophila* homologs of the *IRP* and their targets transferrin and *DMT1* that we induced in the FRDA fly model might confer a protective effect by reducing the increase in cytosolic iron induced by IRP activation that contributes to the mitochondrial iron overload. Taken together, these data demonstrate that modulating the expression of critical genes within key pathways in iron homeostasis is sufficient to suppress FRDA phenotypes in *Drosophila*.

### Knockdown of zinc transporters and copper chaperones ameliorate FRDA fly phenotypes

As we were able to identify suppressors of FRDA phenotypes from our candidate genetic modifier analysis of iron-associated genes, we then evaluated whether pathways also implicated in disease pathogenesis, such as zinc and copper homeostasis [17], may also modify eye and motor performance in the FRDA *Drosophila* model.

**Fig 3:**
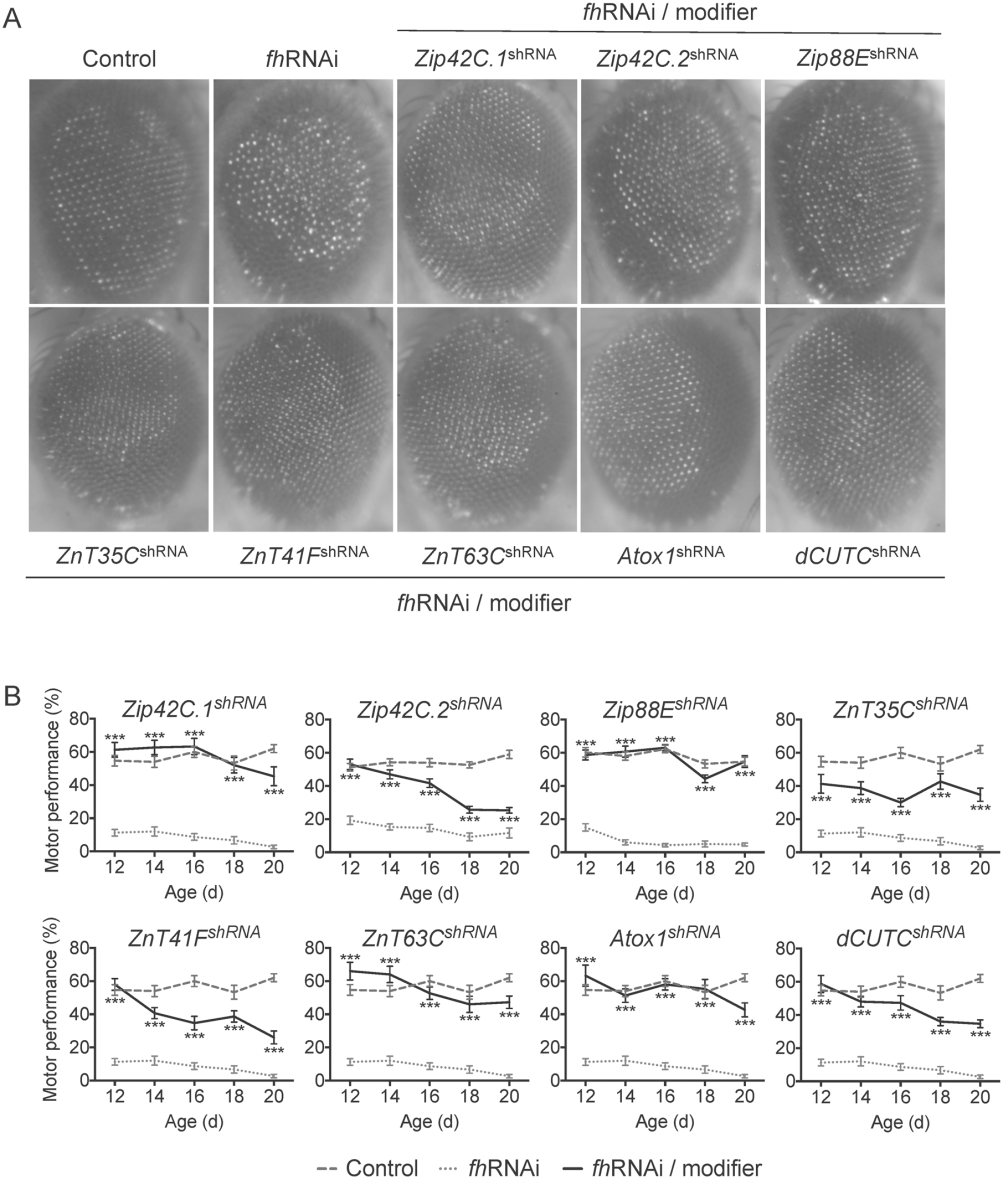
Improvement of the eye morphology and the motor performance phenotypes of FRDA flies by decreased expression of genes implicated in zinc and copper homeostasis. A. Light microscope images of the external eye of control (w^1118^; *GMR*-GAL4), *fh*RNAi (*GMR*-GAL4>UAS-*fh*RNAi-1) and *fh*RNAi/modifier flies (*GMR*-GAL4>UAS-*fh*RNAi-1 additionally carrying the corresponding allele of the modifier) B. Motor performance expressed as the percentage of flies that climbed passed a height of 11.5 cm. after 16 s. was evaluated at different adult ages, from 12 to 20 days old. Control: w^1118^; *actin*-GAL4. *fh*RNAi: *actin*-GAL4>UAS-*fh*RNAi-2. *fh*RNAi/modifier: *actin*-GAL4>UAS-*fh*RNAi-2; modifier allele. The statistical significance between *fh*RNAi and *fh*RNAi/modifier for each day was evaluated by ANOVA followed by Dunnet test for multiple comparisons. *P<0.05, **P<0.01, ***P<0.001. Error bars represent SEM. shRNA: knockdown allele. d: day.

Zinc is essential as a structural or catalytic co-factor in hundreds of proteins such as the zinc finger transcription factors. Zinc transport across membranes is mainly mediated by two conserved gene families of zinc transporters: (1) the Zip family (Zrt-/Irt-like, solute carrier family 39, SLC39A) function in zinc influx from the extracellular medium or vesicular organelles into the cytoplasm, and (2) the ZnT family (Cation diffusion facilitator, CDF, SLC30A) that mediate zinc efflux or compartmentalization. Members of both zinc transporter families have been implicated in neurodegenerative diseases such as AD [42]. Among the orthologous Zip and ZnT transporters in *Drosophila* [43], we found that shRNA against *Zip42C*.*1*, *Zip42C*.*2* and *Zip88E* and *ZnT35C*, *ZnT41F* and *ZnT63C* improved both phenotypes of FRDA model flies (Fig 3A and 3B).

In contrast to zinc, copper is part of a considerably lower number of proteins. However, the mechanisms for regulation of copper uptake, distribution, detoxification and efflux [44], are tightly regulated and evolutionarily conserved due to the redox properties of copper that, when disrupted, may lead to the generation of free radicals. Regarding copper associated-genes, we found that reducing the levels of *Atox1*, a chaperone that delivers copper to ATP7 transporters located in the trans-Golgi network [45], suppressed FRDA phenotypes in *Drosophila* (Fig 3A and 3B). In addition, *dCutC*, a member of the Cut protein family associated with uptake, storage, delivery and efflux of copper [46] was also able to suppress eye degeneration and poor motor performance (Fig 3A and 3B). Taken together, these findings related to both zinc and copper pathways suggest that altering the expression of genes that regulate the balance of metals other than iron may also be beneficial in FRDA.

### MTF-1 overexpression suppress FRDA *Drosophila* model phenotypes

Cells have developed conserved mechanisms to protect themselves from the toxic effects of metals. Under stress conditions, notably metal overload and oxidative stress, the zinc-finger protein Metal-responsive Transcription Factor-1 (MTF-1) translocates to the nucleus and binds to metal response elements (MREs) located in the regulatory regions of its targets genes, such as the metal-sequestering metallothioneins (MT) [47]. MTs are small cysteine rich proteins that bind transition metals with high affinity, thus maintaining low levels of intracellular free metal [48].

**Fig 4:**
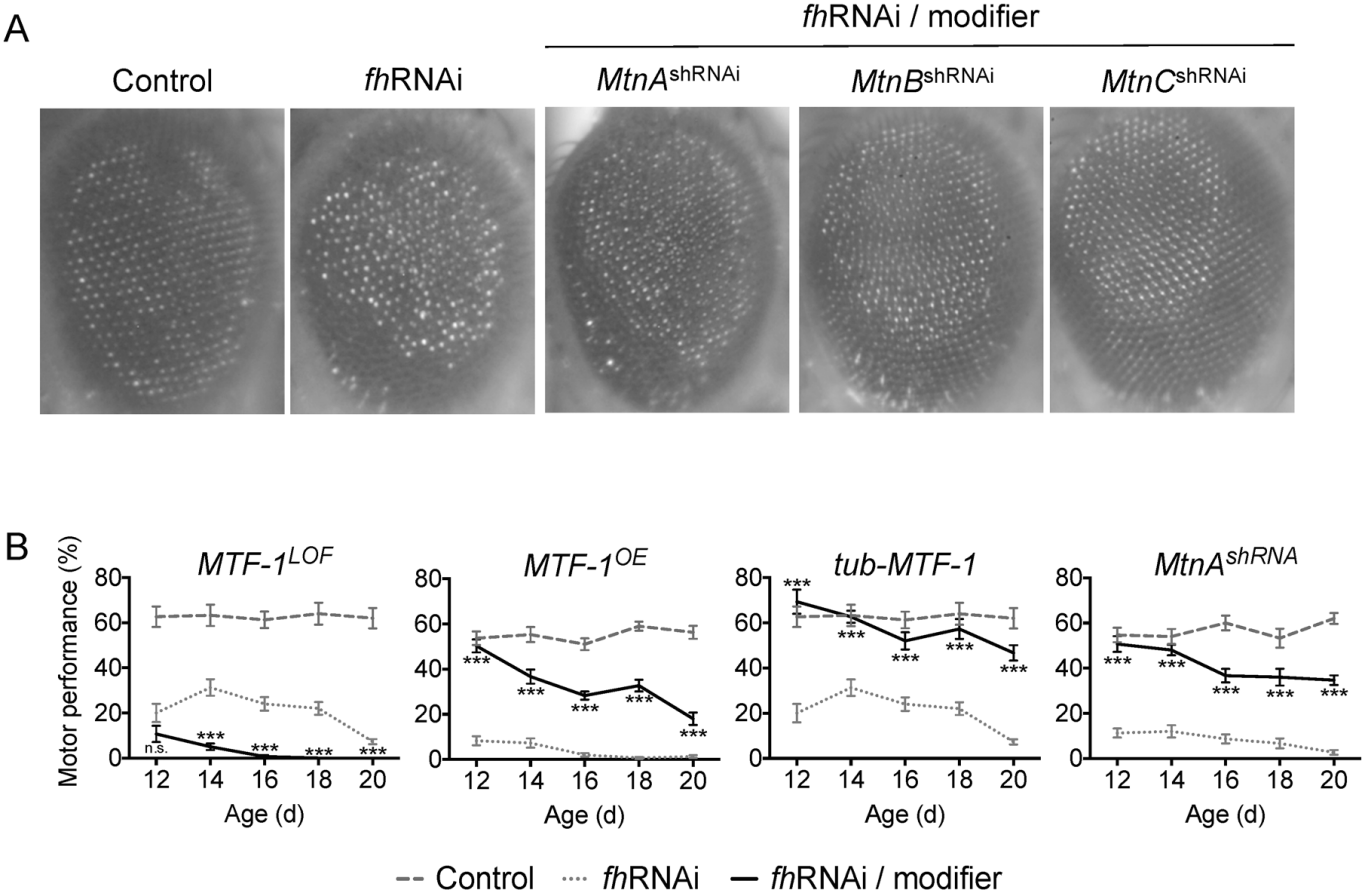
Modification of the eye morphology and the motor performance phenotypes of FRDA flies by altered expression of genes implicated in metal detoxification. A. Light microscope images of the external eye of control (w^1118^; *GMR*-GAL4), *fh*RNAi (*GMR*-GAL4>UAS-*fh*RNAi-1) and *fh*RNAi/modifier flies (*GMR*-GAL4>UAS-*fh*RNAi-1 additionally carrying the corresponding allele of the modifier). B. Motor performance expressed as the percentage of flies that climbed passed a height of 11.5 cm. after 16 s. was evaluated at different adult ages, from 12 to 20 days old. Control: w^1118^; *actin*-GAL4. *fh*RNAi: *actin*-GAL4>UAS-*fh*RNAi-2. *fh*RNAi/modifier: *actin*-GAL4>UAS-*fh*RNAi-2; modifier allele. The statistical significance between *fh*RNAi and *fh*RNAi/modifier for each day was evaluated by ANOVA followed by Dunnet test for multiple comparisons. *P<0.05, **P<0.01, ***P<0.001. Error bars represent SEM. shRNA: knockdown allele. LOF: loss of function allele. OE: overexpression allele. d: day.

In this study we found that MTF-1 is a modifier of FRDA fly model phenotypes, as an enhancer of the motor impairment by loss-of-function and a suppressor when overexpressed (Fig 4B). Similarly, *MTF-1* overexpression in *Drosophila* has been shown to rescue the toxicity induced by oxidative stress [24], the expression of human Aβ42 peptide and a parkin null mutation [49,50]. Contrary to what we expected, knocking down the *Drosophila* MTs, *MtnA*, *MtnB* and *MtnC* (Fig 4A) suppress the eye phenotype of the frataxin deficient animals, as well as the motor performance for *MtnA* (Fig 4B). *MtnB* and *MtnC* knockdown alleles could not be tested for motor performance as their ubiquitous expression driven by *actin*-GAL4 resulted in adult lethality.

To ensure that a dilution of the GAL4 protein was not producing false positive results in the screen, we performed a control experiment testing the expression of the *fh* gene in flies carrying none, one and two UAS constructs. The strains used for this purpose were respectively: (i) the control strain *w*^*1118*^; *actin*-GAL4; (ii) the FRDA model *actin*-GAL4>UAS-*fh*RNAi-2 and (iii) the *actin*-GAL4>UAS-*fh*RNAi-2; UAS-GFP flies carrying a second UAS construct. The expression of *fh* analyzed by RT-qPCR was reduced in the FRDA flies compared to the controls as we had shown in previous works [14,22,25]. In addition, we observed there was no significant difference in *fh* expression levels between the *actin*-GAL4>UAS-*fh*RNAi-2 and the *actin*-GAL4>UAS-*fh*RNAi-2; UAS-GFP lines (S3 Fig). These results demonstrate that the reduction in *fh* expression driven by the UAS-GAL4 system is not affected by the presence of a second UAS construct.

### Suppression of FRDA *Drosophila* model phenotypes is mediated by reducing iron content and oxidative stress

**Fig 5:**
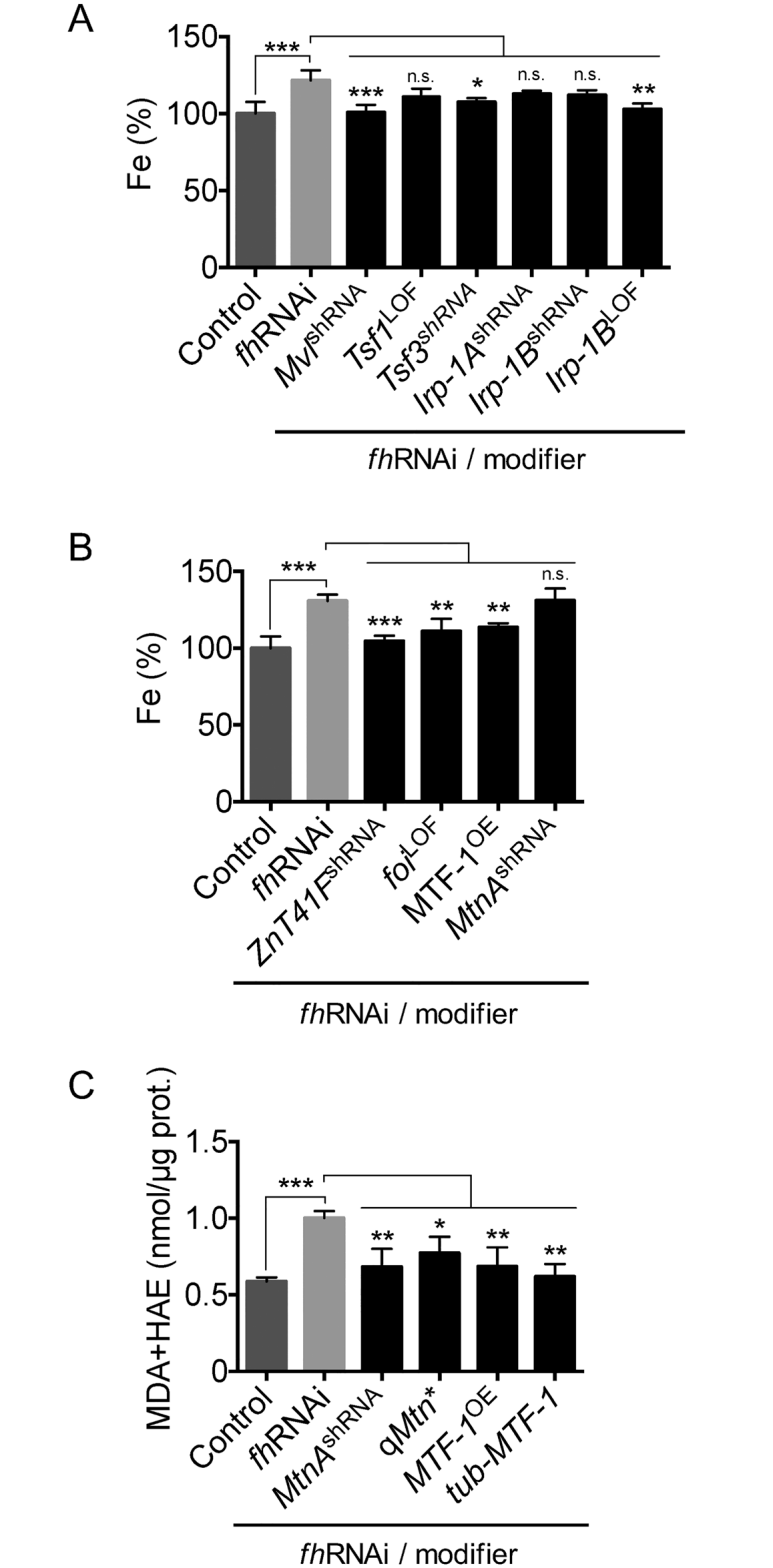
Genetic modifiers of the FRDA phenotypes reduce iron content and oxidative stress. A and B. Increased iron levels are rescued by modification of genes implicated in iron (A) and zinc homeostasis and metal detoxification (B). Total iron content was measured by atomic emission spectroscopy and expressed in percentage relative to the controls. C. Reduction of the increased levels of MDA+HAE by metal detoxification genes. The results are expressed in nmol of MDA+HAE per μg of protein. Error bars represent SEM. Control: w^1118^; *actin*-GAL4. *fh*RNAi: *actin*-GAL4>UAS-*fh*RNAi-2. *fh*RNAi/modifier: *actin*-GAL4>UAS-*fh*RNAi-2; modifier allele. Statistical significance was evaluated by ANOVA followed by Dunnet test for multiple comparisons. n.s.: non-significant, *P<0.05, **P<0.01, ***P<0.001. shRNA: knockdown allele. LOF: loss of function allele. OE: overexpression allele. qMtn*: loss of function of the four metallothioneins MtnA, MtnB, MtnC, MtnD.

We found that altering the expression of genes involved in iron transport, absorption and metabolism ameliorated eye degeneration and motor performance in the FRDA fly model (Fig 2). To determine whether phenotype amelioration was correlated with changes in iron content, we measured the levels of iron in the FRDA model flies expressing the modifier alleles for iron-related genes. We found that three alleles corresponding to suppressors implicated in iron metabolism normalized the levels of this metal as measured by atomic emission spectroscopy, indicating that rescue of FRDA phenotypes can indeed be improved by reducing iron abundance and restoring iron homeostasis (Fig 5A).

In addition, several members of the Zip family have been shown to transport not only zinc but also iron [51–53]. Therefore, we tested whether the improvement in the eye and motor phenotypes in these lines was mediated by a reduction in the iron accumulation displayed by the FRDA flies, independently of their role in zinc transport (Fig 5B). We found that reducing the levels of the Zip transporter *fear-of intimacy* (*foi)* and of *ZnT41F* reduced the iron content in FRDA flies. The loss-of-function of *foi* ameliorated the motor impairment of the FRDA flies (data not shown). According to these results, a reduced expression of at least some of the zinc transporters is sufficient to normalize iron content. We also found that overexpression of *MTF-1* improves the iron accumulation phenotype as well. In contrast, knocking down *MtnA* had no effect on the iron content (Fig 5B).

MTs have been broadly proposed to play a role in antioxidant response [54,55]. However there is evidence indicating that in some cases such as in presence of H_2_O_2_ they can generate hydroxyl radicals [56–58]. To determine the effect of altering the metal detoxification pathway on the levels of oxidative stress of the FRDA flies, we measured the levels of malondialdehyde (MDA) + 4- hydroxyalkenals (HAE). Similar to previous findings [25], we confirmed that the amount of MDA + HAE is indeed higher in *fh*RNAi flies in comparison with controls (Fig 5C). Interestingly, we found that overexpressing *MTF-1* was sufficient to normalize oxidative stress levels (Fig 5C). Reducing the expression of MTs in FRDA flies also improved the oxidative stress phenotype, indirectly supporting their possible role as prooxidants, at least in a frataxin deficient scenario. Taking together the results on the iron and oxidative stress levels, the beneficial effect of overexpressing *MTF-1* may not be mediated by the MTs but most probably through reducing the iron accumulation.

In conclusion, these findings together with the rescue data related to iron-associated suppressors (Fig 2) demonstrate that reducing the iron accumulation through a genetic strategy in FRDA model flies has potential therapeutic benefit. Although direct reduction in iron levels by the use of iron chelators has been only relatively successful in patients [15,16], our results support the notion that improvements in FRDA phenotypes can be mediated directly through pathways regulating iron homeostasis. We also show for the first time that alteration of genes implicated in copper and zinc homeostasis and metal detoxification, as well as copper and zinc chelation also constitute potential therapeutic targets for the disease. Overall, these findings provide the framework for future studies focused on improving metal not only iron homeostasis, either genetically or pharmacologically, in FRDA animal models.

## Supporting Information

S1 Fig Fe levels in the FRDA model flies treated with with the Zn chelator TPEN and the Cu chelators BCS and TTM are not altered. Total iron content was measured using the iron assay kit (BioVision) as we previously reported in [14]. The results are expressed in percentage, taking as 100% the Fe content of flies in the vehicle medium. Error bars represent SEM. The statistical significance between the samples was evaluated by ANOVA followed by Sidak test for multiple comparisons.
(TIF)

S2 Fig Cu,Zn-SOD1 activity after treatment with the Zn chelator TPEN (A) and Cu chelators BCS and TTM (B,C). We found a significant enzyme activity reduction for control and *fh*RNAi-2 flies for BCS and no change for TPEN and TTM. The results are expressed in percentage, taking as 100% the Cu,Zn-SOD1 activity of control flies in the vehicle medium in each assay. Error bars represent SEM. The statistical significance between the samples was evaluated by ANOVA followed by Sidak test for multiple comparisons. *P<0.05, **P<0.01.
(TIF)

S3 Fig *fh* mRNA levels are not altered by the presence of a second UAS construct. Three fly strains carrying different number of UAS constructs were used: *w*^*1118*^; *actin*-GAL4 (control); *actin*-GAL4>UAS-*fh*RNAi-2 (fhRNAi) and *actin*-GAL4>UAS-*fh*RNAi-2;UAS-GFP (*fhRNAi*;UAS_GFP) carrying a second UAS construct. The results are expressed as the fold change of gene expression relative to control levels. Error bars represent SEM. The statistical significance between the samples was evaluated by ANOVA followed by Dunnet test for multiple comparisons. n.s.: non-significant, *P<0.05.
(TIF)

S1 Table Genotypes of the *Drosophila* strains corresponding to genetic interactors implicated in metal homeostasis. (PDF)
