# Supplementary figures and images for: Metal Homeostasis Regulators Suppress FRDA Phenotypes in a Drosophila Model of the Disease
Source: PLoS One. 2016 Jul 19;11(7):e0159209. doi: 10.1371/journal.pone.0159209 (PMC4951068; doi:10.1371/journal.pone.0159209)

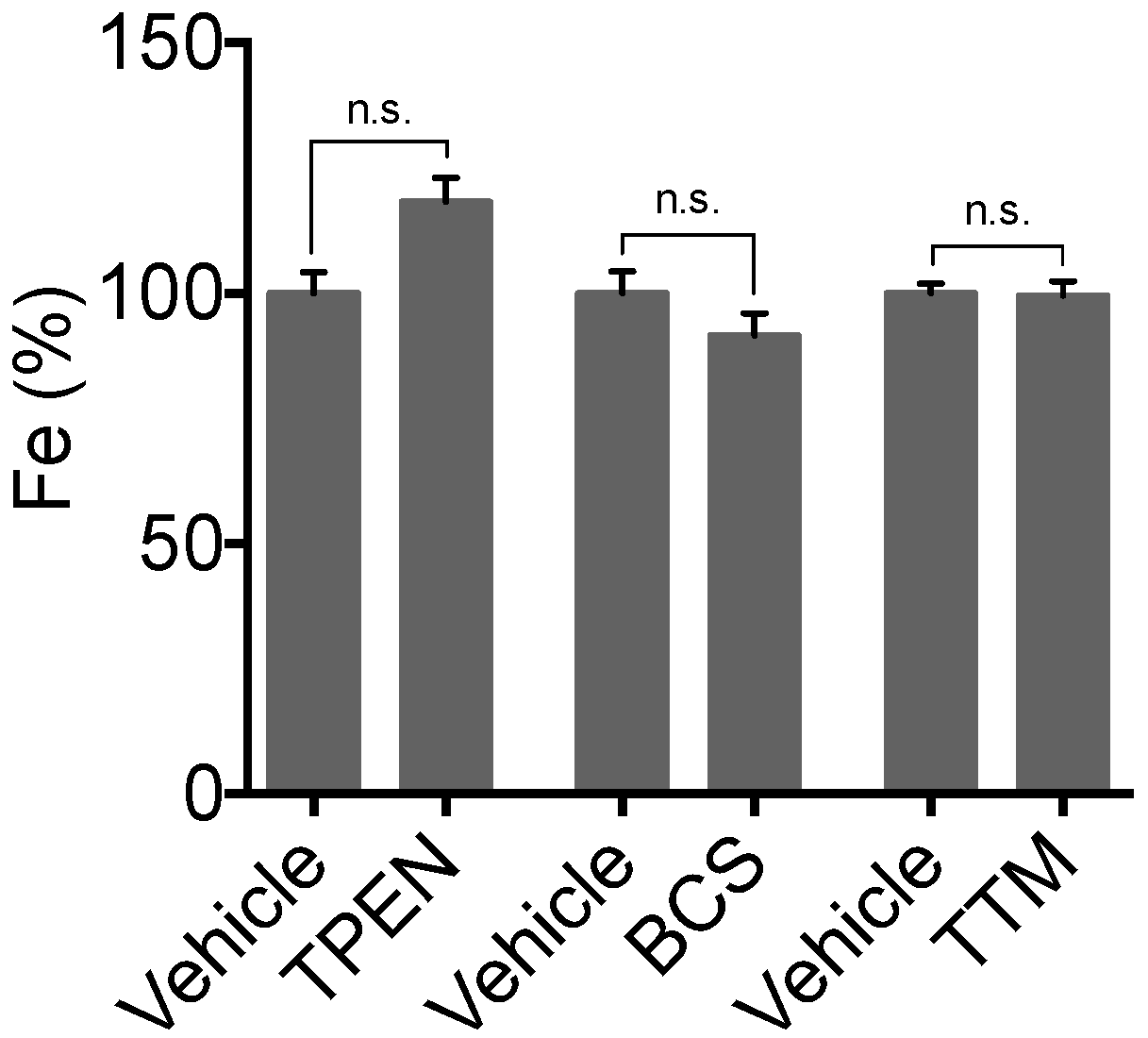

Supplement: S1 Fig — Total iron content was measured using the iron assay kit (BioVision) as we previously reported in [14]. The results are expressed in percentage, taking as 100% the Fe content of flies in the vehicle medium. Error bars represent SEM. The statistical significance between the samples was evaluated by ANOVA followed by Sidak test for multiple comparisons. (TIF) [file pone.0159209.s001.tif]

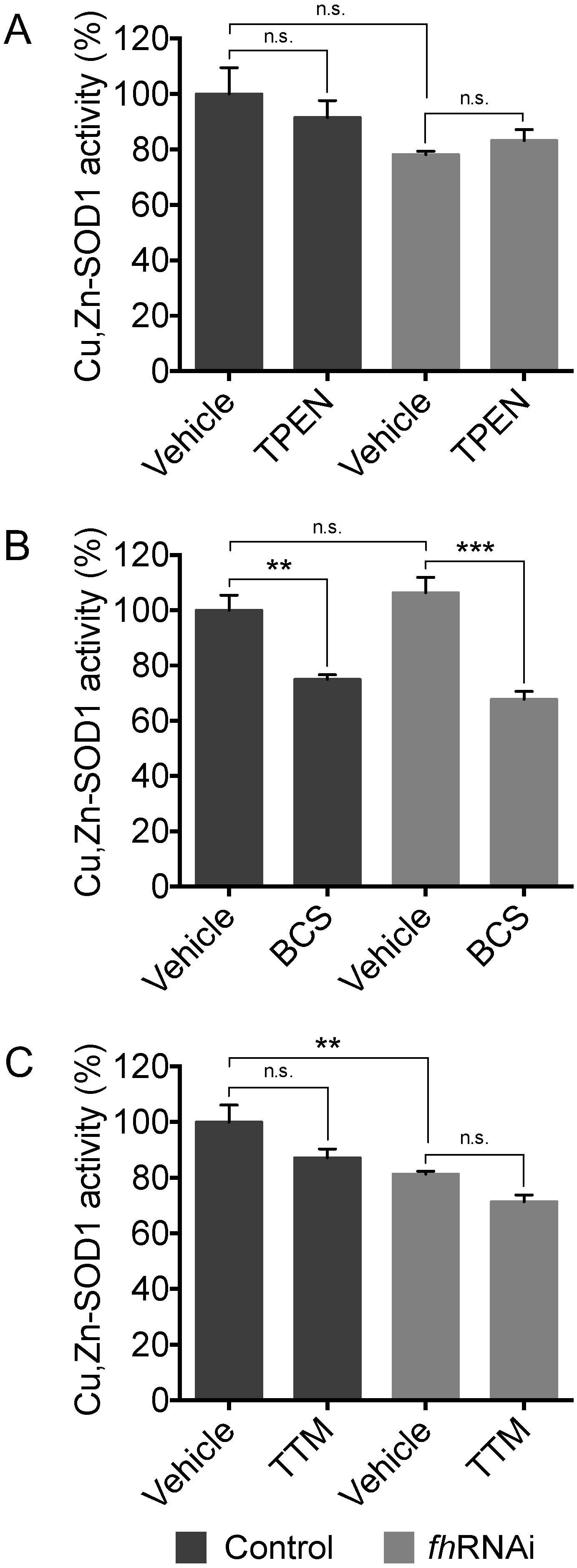

Supplement: S2 Fig — We found a significant enzyme activity reduction for control and fhRNAi-2 flies for BCS and no change for TPEN and TTM. The results are expressed in percentage, taking as 100% the Cu,Zn-SOD1 activity of control flies in the vehicle medium in each assay. Error bars represent SEM. The statistical significance between the samples was evaluated by ANOVA followed by Sidak test for multiple comparisons. *P<0.05, **P<0.01. (TIF) [file pone.0159209.s002.tif]

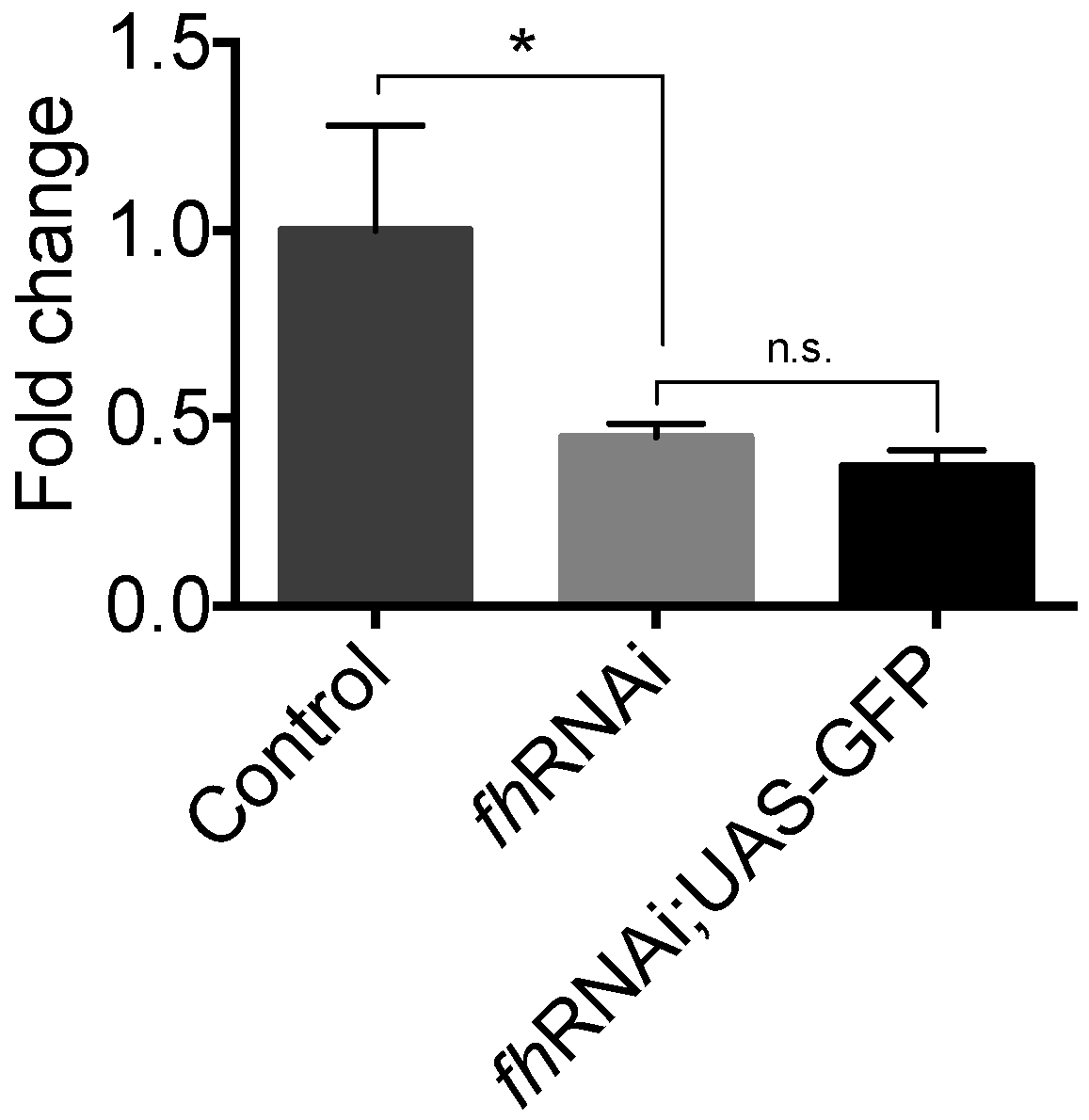

Supplement: S3 Fig — Three fly strains carrying different number of UAS constructs were used: w1118; actin-GAL4 (control); actin-GAL4>UAS-fhRNAi-2 (fhRNAi) and actin-GAL4>UAS-fhRNAi-2;UAS-GFP (fhRNAi;UAS_GFP) carrying a second UAS construct. The results are expressed as the fold change of gene expression relative to control levels. Error bars represent SEM. The statistical significance between the samples was evaluated by ANOVA followed by Dunnet test for multiple comparisons. n.s.: non-significant, *P<0.05. (TIF) [file pone.0159209.s003.tif]
