# Supplementary material for: Metal Homeostasis Regulators Suppress FRDA Phenotypes in a Drosophila Model of the Disease
Source: PLoS One. 2016 Jul 19;11(7):e0159209. doi: 10.1371/journal.pone.0159209 (PMC4951068; doi:10.1371/journal.pone.0159209)
Supplement: S1 Table — (PDF) [file pone.0159209.s004.pdf]

S1 Table. Genotypes of the *Drosophila* strains corresponding to genetic interactors implicated in metal homeostasis.

Strain	Genotype	Effect
Mvl^{shRNA}	P{KK108406}VIE-260B	Knockdown
$Tsf1^{LOF}$	w^{1118} PBac{WH}$Tsf1^{f05108}$	Loss of function
$Tsf1^{shRNA}$	P{KK103815}VIE-260B	Knockdown
$Tsf3^{shRNA}$	P{KK109853}VIE-260B	Knockdown
$Irp\text{-}1A^{shRNA}$	P{KK105828}VIE-260B	Knockdown
$Irp\text{-}1B^{shRNA}$	P{KK108179}VIE-260B	Knockdown
$Irp\text{-}1B^{LOF}$	y^1 w^{67c23}; P{Mae-UAS.6.11}DP00726	Loss of function
$Zip42C.1^{shRNA}$	P{KK108319}VIE-260B	Knockdown
$Zip42C.2^{shRNA}$	P{KK104365}VIE-260B	Knockdown
$Zip88E^{shRNA}$	P{KK110168}VIE-260B	Knockdown
foi^{LOF}	y^1 w*; Mi{MIC}$foi^{MI00610}$/TM3, Sb^1 Ser^1	Loss of function
$ZnT35C^{shRNA}$	P{KK112697}VIE-260B	Knockdown
$ZnT41F^{shRNA}$	P{KK111282}VIE-260B	Knockdown
$ZnT63C^{shRNA}$	P{KK102261}VIE-260B	Knockdown
$Atox1^{shRNA}$	P{KK108811}VIE-260B	Knockdown
$dCutC^{shRNA}$	P{KK108196}VIE-260B	Knockdown
$MtnA^{shRNAi}$	P{KK112833}VIE-260B	Knockdown
$MtnB^{shRNAi}$	P{KK112935}VIE-260B	Knockdown
$MtnC^{shRNAi}$	P{KK114800}VIE-260B	Knockdown
$MTF\text{-}1^{LOF}$	$MTF\text{-}1^{140\text{-}1R}$	Loss of function
$MTF\text{-}1^{OE}$	y^1 w^{67c23}; P{EPgy2}EY03895 [54]	Overexpression
$tub\text{-}MTF\text{-}1^{LOF}$	[24]	Loss of function
q*Mtn**	$MtnA^{\Delta ATG}$ $MtnC^{\Delta ATG}$ $MtnB^{\Delta ATG}$ *MtnD** [55]	Loss of function

Supplementary References

[54] D. Egli, A. Selvaraj, H. Yepiskoposyan, B. Zhang, E. Hafen, O. Georgiev, et al., Knockout of “metal-responsive transcription factor” MTF-1 in Drosophila by homologous recombination reveals its central role in heavy metal homeostasis, EMBO J. 22 (2003) 100–108. doi:10.1093/emboj/cdg012.

[55] D. Egli, J. Domènech, A. Selvaraj, K. Balamurugan, H. Hua, M. Capdevila, et al., The four members of the Drosophila metallothionein family exhibit distinct yet overlapping roles in heavy metal homeostasis and detoxification, Genes Cells. 11 (2006) 647–658. doi:10.1111/j.1365-2443.2006.00971.x.
